# Development and Validation of a Machine Learning Model for the Prediction of Bloodstream Infections in Patients with Hematological Malignancies and Febrile Neutropenia

**DOI:** 10.3390/antibiotics14010013

**Published:** 2024-12-28

**Authors:** Antonio Gallardo-Pizarro, Christian Teijón-Lumbreras, Patricia Monzo-Gallo, Tommaso Francesco Aiello, Mariana Chumbita, Olivier Peyrony, Emmanuelle Gras, Cristina Pitart, Josep Mensa, Jordi Esteve, Alex Soriano, Carolina Garcia-Vidal

**Affiliations:** 1 Department of Infectious Diseases, Hospital Clinic of Barcelona-IDIBAPS, 08036 Barcelona, Spain; agallardo@clinic.cat (A.G.-P.); christian.teijon@gmail.com (C.T.-L.); pmonzom@clinic.cat (P.M.-G.); tfaiello@recerca.clinic.cat (T.F.A.); chumbita@recerca.clinic.cat (M.C.); o.peyrony@hotmail.fr (O.P.); emmgras@gmail.com (E.G.); jmensa@icloud.com (J.M.); asoriano@clinic.cat (A.S.); 2 Facultat de Medicina i Ciències de la Salut, Universitat de Barcelona (UB), c. Casanova, 143, 08036 Barcelona, Spain; 3 Emergency Department, Hôpital Saint-Louis, Assistance Publique-Hôpitaux de Paris, 75010 Paris, France; 4 Institut Pierre Louis d’Épidémiologie et de Santé Publique, Institut National de la Santé et de la Recherche Médicale (INSERM), Sorbonne University, 75012 Paris, France; 5 Department of Microbiology, Hospital Clinic of Barcelona-IDIBAPS, University of Barcelona, 08036 Barcelona, Spain; cristinapitart@ub.edu; 6 Department of Hematology, Hospital Clinic of Barcelona-IDIBAPS, University of Barcelona, 08036 Barcelona, Spain; jesteve@clinic.cat; 7 CIBERINF, CIBER in Infectious Diseases, 28029 Madrid, Spain

**Keywords:** febrile neutropenia, bloodstream infections, hematological malignancies, machine learning, KAMILA

## Abstract

**Background/Objectives:** The rise of multidrug-resistant (MDR) infections demands personalized antibiotic strategies for febrile neutropenia (FN) in hematological malignancies. This study investigates machine learning (ML) for identifying patient profiles with increased susceptibility to bloodstream infections (BSI) during FN onset, aiming to tailor treatment approaches. **Methods:** From January 2020 to June 2022, we used the unsupervised ML algorithm KAMILA to analyze data from hospitalized hematological malignancy patients. Eleven features categorized clinical phenotypes and determined BSI and multidrug-resistant Gram-negative bacilli (MDR-GNB) prevalences at FN onset. Model performance was evaluated with a validation cohort from July 2022 to March 2023. **Results:** Among 462 FN episodes analyzed in the development cohort, 116 (25.1%) had BSIs. KAMILA’s stratification identified three risk clusters: Cluster 1 (low risk), Cluster 2 (intermediate risk), and Cluster 3 (high risk). Cluster 2 (28.4% of episodes) and Cluster 3 (43.7%) exhibited higher BSI rates of 26.7% and 37.6% and GNB BSI rates of 13.4% and 19.3%, respectively. Cluster 3 had a higher incidence of MDR-GNB BSIs, accounting for 75% of all MDR-GNB BSIs. Cluster 1 (27.9% of episodes) showed a lower BSI risk (<1%) with no GNB infections. Validation cohort results were similar: Cluster 3 had a BSI rate of 38.1%, including 78% of all MDR-GNB BSIs, while Cluster 1 had no GNB-related BSIs. **Conclusions:** Unsupervised ML-based risk stratification enhances evidence-driven decision-making for empiric antibiotic therapies at FN onset, crucial in an era of rising multi-drug resistance.

## 1. Introduction

Bloodstream infections (BSIs) present a significant risk for neutropenic patients with hematological malignancies, primarily because of their high morbidity and mortality [1]. The underlying pathophysiology of these malignancies—characterized by disease-induced immunosuppression, chemotherapy-induced neutropenia, and mucosal damage—predisposes patients to severe infections [2]. Among this population, BSIs occur in 10–25% of cases, escalating to 55% in those undergoing hematopoietic stem cell transplantation (HSCT) [3,4,5]. These infections are a major driver of treatment delays, prolonged hospital stays, and mortality rates ranging from 12% to 42%, depending on the pathogen and resistance profiles [6,7,8].

In response, current guidelines emphasize the immediate use of broad-spectrum antibiotics during febrile neutropenia (FN) [9,10]. However, despite these guidelines, the rise in multidrug-resistant (MDR) bacteria has led to alarming rates of inappropriate empirical antibiotic therapy in this population, following existing protocols [11,12,13]. Nowadays this problem is a major challenge due to a high number of Gram-negative bacilli (GNB) infections being caused by MDR strains [3,14]. These therapeutic errors in patients with BSI have led to increased mortality [15,16]. The optimal strategy to mitigate these misguided treatments remains undefined; introducing wider-spectrum and new antibiotics for all patients may not be feasible, and the combination of traditional antibiotics in all neutropenic patients has not proven to improve outcomes and could result in increased toxicity [17,18].

The economic burden of BSIs is substantial, with increased ICU admission rates, extended antimicrobial therapy, and rising costs associated with resistance management [19]. Prevention and precision in management are therefore critical. Infection control measures, such as antimicrobial stewardship programs and surveillance cultures, have demonstrated efficacy in reducing MDR infections and guiding appropriate therapy [20,21,22]. However, gaps remain in the ability to stratify patients based on BSI risk at FN onset, delaying the initiation of optimal therapies.

In the current era, where artificial intelligence (AI) is being integrated into global healthcare, there is an opportunity for more refined risk stratification and specifically tailored treatments [23,24]. Machine learning (ML) models have demonstrated superiority over traditional methods in predicting complications in patients with FN while minimizing subjectivity in clinical decisions and addressing the limitations of existing clinical scales [25]. Our study aimed to assess the potential role of the KAy-means for MIxed LArge data (KAMILA) algorithm, an unsupervised ML method [26], in distinguishing BSI risks among different clinical phenotypes at FN onset in patients with hematological malignancies. This approach may provide essential insights into the development of personalized antibiotic strategies for these patients.

## 2. Results

### 2.1. Patient Characteristics and Description of BSI

The development cohort population consisted of 331 patients diagnosed with hematological malignancies, who had 462 episodes of FN during the study period. A BSI was identified in 116, accounting for 25.1%. Tables S1 and S2 detailed the BSI and other bacterial infections, respectively. GNB were implicated in 57/116 (49.1%) of BSI, while MDR-GNB were identified in 24 episodes (20.7% of all episodes within the cohort; 42.1% among GNB).

Table S3 showed all patient data from the development cohort, categorized according to BSI status. The comparative characteristics of the features introduced into the clustering model are detailed by group in Table 1.

### 2.2. Findings Derived Using the KAMILA Algorithm

The KAMILA algorithm was evaluated using configurations of three, four, and five clusters. The three-cluster model was selected through the prediction strength criterion and a clinical validation approach to balance the model’s fit with its complexity. Within this model, Cluster 2 (intermediate-risk) included 131 episodes (28.4% of the development cohort), of whom 35/131 (26.7%) had BSI; Cluster 3 (high-risk) included 202 (43.7%) episodes, 76/202 (37.6%) with BSI; and Cluster 1 (low-risk) included 129 (27.9%) episodes, 5/129 (3.9%) with BSI, and 1/129 (0.8%) case remaining after excluding CoNS BSI.

**Figure 1:**
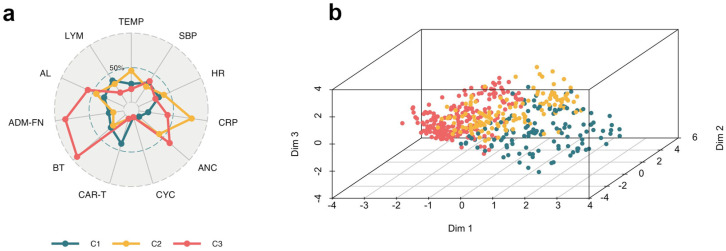
Insights from cluster analysis for three-cluster solution: (**a**) Radar chart showing the mean values on the scaled axes; (**b**) Scatter plot of the three-cluster solution on the FAMD’s first three dimensions. All variables, including numerical variables, were converted to a binary format and coded numerically. C refers to clusters with values ranging from 1 to 3. Abbreviations: ADM-FN, time from admission to onset of febrile neutropenia; AL, acute leukemia; ANC, absolute neutrophil count (<100 cells/µL); BT, blood transfusion; CAR-T, chimeric antigen receptor T-cell therapy; CRP, C-reactive protein (>10 mg/dL); CYC, cyclophosphamide; Dim, dimension; FAMD, factor analysis for mixed data; HR, heart rate (>110 bpm); LYM, lymphoma; SBP, systolic blood pressure (<95 mmHg); TEMP, temperature (>38.5 °C).

A total of 24/462 (5.2%) of MDR-GNB BSIs were documented in our cohort. Their distribution across clusters was 6/131 episodes (4.6%) in Cluster 2, 18/202 episodes (8.9%) in Cluster 3, and 0/129 episodes (0%) in Cluster 1. Cross-cluster clinical differences are illustrated using the radar plot shown in Figure 1a. The first three components of FAMD were employed to shape the cluster patterns, as illustrated in Figure 1b.

#### Three-Cluster Solution Interpretation

**Figure 2:**
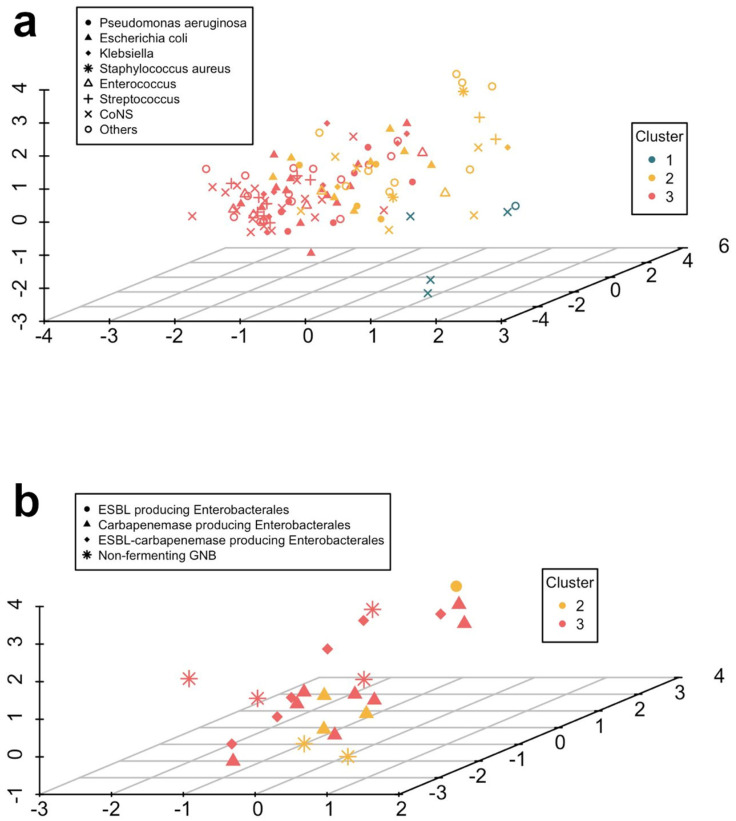
Etiology of isolates across three clusters in the derivation cohort: (**a**) Bloodstream Infections and (**b**) multidrug-resistant Gram-negative bacilli. Scatter plots of the three-cluster solution on the first three dimensions of FAMD were employed for the visualization. Cluster 1 is not represented in panel b, as there were no isolates of these pathogens in this cluster. Abbreviations: BSI, bloodstream infection; CoNS, coagulase-negative Staphylococci; ESBL, extended-spectrum beta-lactamases; FAMD, factor analysis for mixed data; GNB, Gram-negative bacilli; MDR, multidrug-resistant.

Cluster 1, characterized by its low BSI risk profile, had four BSI cases related to CoNS and a single case associated with *Rothia mucilaginosa.* This cluster did not exhibit any BSI attributed to susceptible or resistant Enterobacterales or *Pseudomonas aeruginosa*. In contrast, clusters 2 and 3 displayed a heightened BSI risk, as evidenced by a hazard ratio (HR) of 6.64 (95% confidence interval [CI]: 2.68 to 16.43; *p* = 0.001) and HR of 8.95 (95% CI: 3.15 to 20.55; *p* < 0.001), respectively. Figure 2a provides a systematic depiction of the etiology of BSI across the three clusters. It is important to note that Cluster 3 was also identified as the most vulnerable to MDR-GNB-related BSI, reaching a rate of 8.9% of all episodes within this cluster. Specifically, it constitutes 46.2% of all GNB BSI within this cluster and represents 75% of all MDR-GNB BSI in the development cohort. Figure 2b shows the distribution of MDR-GNB isolates per cluster, and Table S4 provides detailed enumerations of all such isolates.

Table 2 presents a comparative analysis of the prevalence of categorical features and the associated lift values. In intermediate-risk Cluster 2, distinctive features included a higher occurrence of body temperature exceeding 38.5 °C, a heart rate surpassing 110 beats per minute, and CRP levels exceeding 10 mg/dL. Those patients mainly had a few days from hospital admission to fever onset. In high-risk Cluster 3, a high prevalence of acute leukemia and frequent use of cyclophosphamide or blood transfusions were observed. Additionally, patients in this cluster more frequently presented with an SBP less than 95 mmHg and experienced extended hospital stays until the onset of FN. Finally, within low-risk Cluster 1, patients were frequently diagnosed with lymphoma and underwent CAR-T therapy. It was uncommon for these patients to exhibit an ANC less than 0.1 × 10^3^/µL. Figure S1 provides a detailed comparison of continuous features among the clusters.

### 2.3. Cluster Analysis Outcomes in the Validation Cohort

The predictive model was applied to determine the cluster assignments of the 91 episodes of FN within the validation cohort. Table 3 compares the most important features between the development and validation cohorts.

**Figure 3:**
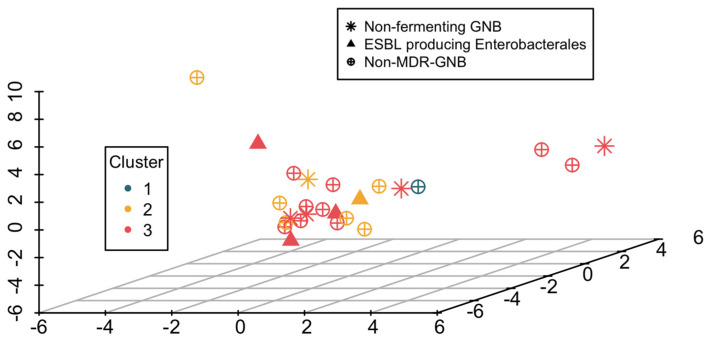
Etiology of isolates across three clusters in the validation cohort. Scatter plot of the three-cluster solution on the first three dimensions of FAMD was employed for the visualization. Abbreviations: BSI, bloodstream infection; CoNS, coagulase-negative Staphylococci; ESBL, extended-spectrum beta-lactamases; GNB, Gram-negative bacilli; MDR, multidrug.

In the validation cohort, Cluster 2 included 32 episodes (35.1%), with 8/32 (25.0%) showing BSI; Cluster 3 comprised 42 episodes (46.2%), with 16/42 (38.1%) experiencing BSI; and Cluster 1 contained 17 episodes (18.7%), with 1/17 (5.9%) having BSI. This single case in Cluster 1 was an *Enterococcus faecium* BSI. Figure 3 illustrates the etiology of BSIs across the three identified clusters in the validation cohort. Table S5 contains a complete list of microbiologically documented bacterial BSIs from the validation cohort, while Table S6 details the MDR-GNB BSI. The incidence of MDR-GNB BSIs was 6.3% (2/32 episodes) in Cluster 2 and 16.7% (7/42 episodes) in Cluster 3, with 77.8% of all MDR-GNB BSIs in the validation cohort occurring in Cluster 3.

## 3. Discussion

Our study documented that hematological patients with malignancies experiencing FN can be categorized into three distinct clusters based on their clinical phenotypes, each representing a different risk level for developing a BSI and MDR-GNB BSI. Patients in the low-risk cluster exhibited clinical characteristics that elucidated model outcomes. First, they were predominantly individuals with lymphoma (42%) and had shorter periods of neutropenia. Consequently, an endogenous BSI was less common. Second, most episodes had the highest neutrophil counts (even below 0.5 × 10^3^/µL), and some were in a phase of neutrophil recovery, potentially causing fever by themselves. Finally, this group included patients who had recently received CAR-T cell therapy infusions (40%) and may have experienced inflammatory fever [27]. Conversely, our model classifies patients at higher risk of BSIs as those with acute leukemia (58%) and profound neutropenia, who have undergone cyclophosphamide treatment (45%), exhibit greater hypotension, and experience FN episodes after prolonged hospital stays. The use of cyclophosphamide in the context of post-transplant prophylaxis (PTCY) has been associated with an increased incidence of BSIs, particularly during the early post-transplant phase. These infections are observed in nearly half of PTCY patients compared to less than a third of those receiving other prophylaxis, linked to delayed immune reconstitution, prolonged neutropenia, gut dysbiosis, the need for ICU admission, and extended hospitalization [5,28]. This suggests that they are at an increased risk of endogenous bacteremia and catheter-related infections, the two most common sources of infection in this population, and identify those with more septic clinical parameters [29]. This population, with prolonged hospitalizations, is also more prone to having MDR-GNB infections.

In this framework, FN episodes can be distinguished into clusters with varying BSI risks: one cluster with a very low risk and two with higher risks. Importantly, within the cluster identified as low risk, no bacteremia due to GNB was observed. This differentiation could be key to selecting a personalized empirical antibiotic treatment. The use of clustering techniques and/or AI has led to a revolution in personalizing clinical decision-making processes [30]. Existing literature utilizing ML methodologies has predominantly pinpointed elevated BSI risks within selected cohorts, namely pediatric populations and HSCT recipients [24,25,31,32]. In our methodological approach, we integrated a risk stratification tool based on the KAMILA algorithm, as it allows for handling both continuous and categorical variables [33].

The greatest applicability of objectively categorizing patients with different risks of BSIs lies in the potential to administer personalized empirical antibiotic treatments, with the main objective of diminishing inappropriate antibiotic use in bacteremia neutropenic patients while making a policy of rationalization of antibiotic consumption. Current FN guidelines advocate a uniform empirical antibiotic approach for most patients with FN [9,10]. A significant challenge arises due to the increasing prevalence of antibiotic-resistant GNB, particularly Enterobacterales and *Pseudomonas aeruginosa*. It has been recently demonstrated that the antibiotics currently recommended in guidelines are often inappropriate, and inadequate empirical treatment, especially in patients with GNB bacteremia, is associated with higher mortality rates [11,12,13,16]. Furthermore, various studies indicate that combined antibiotic treatments do not benefit all patients with neutropenic fever but may be advantageous for those who ultimately develop bacteremia due to GNB [13,16,17,34]. Thus, accurately predicting which patients will develop bacteremia could optimize the selection of those who would benefit from combined antibiotic regimens.

Our study has several limitations. Firstly, as this was a single-center investigation with specific guidelines for infection prophylaxis and prevention, there is an inherent need for external validation. Secondly, although our study included one of the largest numbers of FN episodes reported in the literature, the number of episodes remains relatively small in studies employing ML-based methodologies. Our strategy prioritizes data quality, yet our methodology would benefit from learning from a larger number of events [30]. To ensure the consistency and reliability of our findings, we performed validation using a cohort derived from the same center. Finally, it is noteworthy that the global epidemiology and prevalence of MDR-GNB can vary significantly across regions, potentially leading to variations in risk stratification.

Our results pave the way for a new approach to treatment personalization. Among the patients classified in the lower-risk cluster for BSI, constituting 26% of the total patient cohort, none would have significantly benefited from any of the broad-spectrum treatments recommended by the current guidelines. None of the patients experienced bacteremia or other positive bacterial cultures that required anti-pseudomonal beta-lactams. One option could involve recommending ceftriaxone or levofloxacin to this low-risk population and/or delaying the initiation of empirical antibiotic treatment. In contrast, those classified in the higher-risk group, which encompasses the majority of patients with MDR-GNB bacteremia, accounting for 75% of all MDR-GNB BSI, could potentially be empirically treated with newer beta-lactam and broader-spectrum agents to avoid inappropriate treatments. The intermediate-risk group may adhere to an antibiotic approach similar to that recommended by current treatment guidelines. Our study demonstrates that risk stratification for bacteremia within this population is possible. However, the implications of different empirical antibiotic strategies should be scrutinized in future studies.

## 4. Materials and Methods

### 4.1. Setting, Study Population, and Study Design

This retrospective observational cohort study was conducted at the Hospital Clinic in Barcelona, Spain, a 700-bed university-affiliated hospital serving an urban catchment area of over 500,000 adults. Two separate cohorts were utilized for the study. The development cohort comprised all FN episodes from adult patients with hematological malignancies admitted between January 2020 and June 2022. This cohort was used to build the ML model. The validation cohort, consisting of FN episodes from July 2022 to March 2023, was used to assess the model’s performance and generalizability. High-quality data on demographic characteristics, clinical vital signs, laboratory findings, microbiological test results, therapeutic interventions, and patient outcomes were extracted from electronic health records as previously described [30]. Data quality was assessed by a multidisciplinary team that included physicians and data scientists.

### 4.2. Definitions

Patients with FN were defined as those who had a temperature measurement greater than 38.0 °C and an absolute neutrophil count (ANC) of less than 0.5 × 10^3^/µL [9]. Separate episodes of FN were considered to be those whose febrile determination was preceded by more than 5 days of apyrexia.

A BSI was defined as the growth of non-skin commensal flora in one or more blood cultures. To define bacteremia caused by a common skin colonizer, such as coagulase-negative Staphylococci (CoNS) or *Corynebacterium* spp., two or more positive blood cultures were required. These cultures must have been drawn from different sites and have demonstrated the same susceptibility pattern [35]. Positive cultures were considered to be related to FN events when collected within 24 h of FN onset. In accordance with hospital protocols, patients with expected neutropenia for more than 10 days received azoles and/or fluoroquinolone prophylaxis.

The following GNB were classified as MDR: (i) ESBL-producing Enterobacterales, (ii) carbapenemase-producing Enterobacterales, and (iii) non-fermenting GNB resistant to at least one antibiotic in three classes of antibiotics: carbapenems, ureidopenicillins, cephalosporins (ceftazidime and cefepime), monobactams, aminoglycosides, and fluoroquinolones [36].

### 4.3. Microbiological Methods

Our center follows international guideline recommendations to collect and incubate cultures [37]. Blood samples were processed using the BACTEC FX system (Becton Dickinson Microbiology Systems, BD, Franklin Lakes, NJ, USA) and incubated for 5 days. Isolates were identified using conventional microbiological techniques. Antimicrobial susceptibility testing was performed using either an automated microdilution system (Phoenix system, Becton, Dickinson and Company, Franklin Lakes, NJ, USA) or a gradient strip Etest (AB BIODISH, Solna, Sweden/bioMérieux, Marcy-l’Etoile, France). ESBLs were suspected based on minimum inhibitory concentration results and confirmed by double-disc synergy testing using discs containing cefotaxime, ceftazidime, and cefepime, which were applied to plates next to a disc containing clavulanic acid [38]. Carbapenemase-producing Enterobacterales were phenotypically detected using a modified carbapenem inactivation method [39]. We adhered to the European Committee on Antimicrobial Susceptibility Testing breakpoints applicable for each year to classify isolates as either susceptible or resistant; isolates with intermediate susceptibility were categorized as resistant [40].

### 4.4. Statistical Analysis, Model Development, and Validation

#### 4.4.1. Cohort Analysis

Descriptive statistics for the entire cohort included median values with interquartile ranges (IQRs) for continuous variables, while categorical variables were described using absolute numbers and percentages. Categorical variables were compared using either the chi-squared (χ^2^) test or Fisher’s exact test when appropriate, and continuous variables were compared using the Mann–Whitney U test. The significance threshold was set at *p* < 0.05. Data analysis during the study was performed using RStudio version 4.2 (RStudio Inc., Boston, MA, USA) with the following packages: (1) *mice*, (2) *kamila*, (3) *factoextra*, (4) *survival*, (5) *scatterplot3d*, (6) *ggradar*, (7) *ggplot*, and (8) *plotly* [41,42,43,44,45,46,47].

#### 4.4.2. Application of the Clustering Workflow and Data Preparation

Our methodology strictly adhered to the Transparent Reporting of a Multivariable Prediction Model for Individual Prognosis or Diagnosis (TRIPOD) standards [48]. We integrated statistically significant features from the univariate analyses into our model. For the development of the ML algorithm, we excluded BSIs attributed to CoNS given their special characteristics [49].

To enhance the robustness of our model, we excluded categorical variables with low prevalence—specifically, those present in fewer than 10% of cases. This approach aligns with best practices for sensitive statistical methods such as Multiple Correspondence Analysis (MCA), as supported by previous research [50]. No missing data were identified for categorical variables. Continuous variables with over 30% missing data were excluded from the clustering process [51]. Missing values were imputed using the predictive mean-matching method [41]. Five imputed datasets were generated, and their mean values calculated and rounded to replace the missing data. Continuous variables were standardized by centering them to a mean of 0 and standard deviation of 1.

#### 4.4.3. KAMILA Clustering

We employed the KAMILA algorithm, implemented in the R package kamila (version 0.1.2, released 10 March 2020) [26,42]. This method combines kernel density estimation for continuous variables and multinomial modeling for categorical variables. KAMILA balances the contributions of both variable types and is suitable for large and heterogeneous datasets. Its robustness and accuracy have been validated in simulated and clinical scenarios, outperforming other methods for mixed-type data [52,53]. Further details are available on CRAN (https://CRAN.R-project.org/package=kamila, accessed on 10 December 2024).

In the development of our predictive model, we incorporated features from five key domains: (i) hematological disease, encompassing lymphoma and acute leukemia; (ii) clinical vital signs, such as temperature, systolic blood pressure (SBP), and heart rate; (iii) laboratory findings, including C-reactive protein (CRP) and ANC; (iv) therapeutic interventions, accounting for chimeric antigen receptor T-cell (CART), the use of cyclophosphamide or regimens including it since admission, and blood transfusions recorded from admission to the episode; and (v) temporal hospital stay metrics, concentrating on the period from admission to the onset of FN. The KAMILA algorithm was executed with 300 random initializations and a limit of 25 iterations for each initialization. The optimal number of clusters was determined through a clinical validation approach, supported by the prediction strength criterion and using independent cohorts for model evaluation and validation. This method balances the model’s fit with its complexity, ensuring that the model performs reliably in real clinical settings [54,55]. To address the complexities of visualizing multidimensional data, we used factor analysis for mixed data (FAMD), a technique that integrates principal component analysis (PCA) for numeric variables with MCA for categorical ones [56].

#### 4.4.4. Analysis of Cluster Differences and BSI-Related Events

We harnessed the features employed during the clustering phase to compute comprehensive descriptive statistics encompassing both proportions and lift values. The lift represents the ratio of representation within the cluster to that of the entire cohort. Continuous variables were initially categorized based on their median values and then adjusted to a more appropriate threshold to enhance their clinical interpretation. This approach facilitates a comparison between clusters. To evaluate variations in BSI-related events across clusters, we employed Cox regression.

## 5. Conclusions

The implementation of the KAMILA algorithm achieved precise risk stratification for a BSI and MDR BSI at the onset of FN in patients with hematological malignancies. Importantly, the results of this clustering technique can be interpreted from a clinical perspective.

Our study highlights the potential of this methodology to enhance objective decision-making in the selection of empirical antibiotic therapies. This finding is particularly significant given the increasing challenges posed by multidrug resistance. The current high incidence of inappropriate empirical antibiotic use, even when adhering to major guidelines, is unacceptable, and it has been associated with increased mortality.

Identifying strategies to personalize antibiotic treatments and provide each patient with the necessary spectrum of coverage is crucial. Using our algorithm, hematological patients with malignancies can be stratified into three distinct groups: one with no major risk of GNB infection; a second group with a BSI rate of approximately 25%, where most BSIs are caused by GNBs susceptible to the majority of antibiotics; and a third group characterized by a high BSI rate (approaching 40%) and a significant burden of MDR-GNB infections. This final group represents patients who would require broader-spectrum empirical antibiotic therapy.

Further research is required to validate the generalizability of these findings to various clinical settings and to determine the impact of the model on patient outcomes, antibiotic stewardship, and healthcare costs.

## Figures and Tables

**Table 1:** Comparative characteristics stratified by bloodstream infection status and bloodstream infection excluding coagulase-negative Staphylococci afterward in the development cohort.

Domains	Bloodstream Infection *					Non-CoNS Bloodstream Infection **					Missing Data
N	Yes		No		*p*-Value	Yes		No		*p*-Value	
	116		346			89		373			
**Haematological disease**, n (%)											
Lymphoma	19	(16.4)	112	(32.4)	0.001	17	(19.1)	114	(30.6)	0.04	0
Acute leukemia	71	(61.2)	147	(42.5)	<0.001	51	(57.3)	167	(44.8)	0.04	0
**Clinical vital signs**, median [IQR]											
Temperature (°C)	38.3	[38.1–38.7]	38.3	[38.1–38.6]	0.07	38.4	[38.1–38.8]	38.2	[38.1–38.6]	0.02	0
Heart rate (bpm)	103	[90–120]	102	[93–114]	0.44	105	[95–121]	101	[92–113]	0.02	0
SBP (mmHg)	97	[85.5–107]	100	[92–109]	0.04	97	[85–107.5]	100	[92–109]	0.03	5
**Laboratory findings**, median [IQR]											
Neutrophil count [cells/mm^3^]	0	[0–0.1]	0.1	[0–0.3]	<0.001	0	[0–0.1]	0.1	[0–0.3]	<0.001	0
CRP [mg/dL]	9.1	[4.4–17.8]	7.9	[3.4–13.9]	0.14	9.2	[5.1–17.8]	7.8	[3.4–13.9]	0.04	112
**Therapeutic interventions**, n (%)											
Cyclophosphamide ^a^	47	(40.5)	80	(23.1)	<0.001	40	(44.9)	87	(23.3)	<0.001	0
CAR-T ^a^	2	(1.7)	56	(16.2)	<0.001	0	(0)	58	(15.5)	<0.001	0
Blood transfusion ^a^	76	(65.5)	180	(52.0)	0.02	62	(69.7)	194	(52.0)	0.01	0
**Temporal hospital stays metrics**, median [IQR]											
Admission to onset of FN [days]	15	[7.8–21.2]	8	[2–16]	<0.001	16	[7–22]	9	[2–16]	<0.001	0

* Two cases of fungemia caused by *Candida glabrata* and *Candida albicans* were included in the analysis. ** For the purpose of this comparison, bacteremia caused by CoNS was reassigned to the non-bloodstream infection group. ^a^ Administered from admission to episode start. Abbreviations: bpm, beats per minute; CAR-T, chimeric antigen receptor T-cell; CoNS, coagulase-negative Staphylococci; CRP, C-reactive protein; FN, febrile neutropenia; SBP, systolic blood pressure.

**Table 2:** Comparative analysis of bloodstream infection rates, categorical features prevalence, and associated lift values in the three-cluster solution of the KAMILA model in the development cohort.

		C1		C2		C3		Development Cohort
	N/(%)	129/(27.9)		131/(28.4)		202/(43.7)		462/(100)
Outcomes	**Bloodstream infection**, number of cases/rates (%) and lift values							
	Bloodstream infection	5/(3.9)	0.16	35/(26.7)	1.06	76/(37.6)	1.50	116/(25.1)
	Bloodstream infection excluding CoNS	1/(0.8)	0.04	29/(22.1)	1.15	59/(29.2)	1.51	89/(19.3)
	MDR related bloodstream infection	0/(0)	0	7/(5.3)	0.95	19/(9.4)	1.68	26/(5.6)
	MDR-GNB related bloodstream infection	0/(0)	0	6/(4.6)	0.88	18/(8.9)	1.71	24/(5.2)
Categorical features	**Haematological disease**, number of cases/prevalence (%) and lift values							
	Lymphoma	54/(41.9)	1.47	42/(32.1)	1.13	35/(17.3)	0.61	131/(28.4)
	Acute leukemia	39/(30.2)	0.64	62/(47.3)	1	117/(57.9)	1.23	218/(47.2)
	**Clinical vital signs**, number of cases/prevalence (%) and lift values							
	Temperature > 38.5 °C	30/(23.3)	0.82	68/(51.9)	1.81	34/(16.8)	0.59	132/(28.6)
	Heart rate > 110 bpm	41/(31.8)	0.97	59/(45.0)	1.38	51/(25.2)	0.77	151/(32.7)
	SBP < 95 mmHg	45/(34.9)	1	37/(28.2)	0.81	79/(39.1)	1.12	161/(34.8)
	**Laboratory findings**, number of cases/prevalence (%) and lift values							
	Neutrophil count < 0.1 × 10^3^/µL	6/(4.7)	0.11	54/(41.2)	1.01	129/(63.9)	1.56	189/(40.9)
	CRP > 10 mg/dL	18/(14.0)	0.33	99/(75.6)	1.78	79/(39.1)	0.92	196/(42.4)
	**Therapeutic interventions**, number of cases/prevalence (%) and lift values							
	Cyclophosphamide ^a^	29/(22.5)	0.82	7/(5.3)	0.19	91/(45.0)	1.64	127/(27.5)
	CAR-T ^a^	52/(40.3)	3.20	1/(0.8)	0.06	5/(2.5)	0.20	58/(12.6)
	Blood transfusion ^a^	37/(28.7)	0.52	36/(27.5)	0.50	183/(90.6)	1.64	256/(55.4)
	**Temporal hospital stays metrics**, number of cases/prevalence (%) and lift values							
	Admission to onset of FN > 10 days	28/(21.7)	0.45	21/(16.0)	0.33	173/(85.6)	1.78	222/(48.1)

^a^ Administered from admission to episode start. C1, C2, and C3 stand for cluster 1, cluster 2, and cluster 3, respectively. Light blue: ≤0.5; blue: ≤1.0; yellow: ≤1.5; and green: >1.5. Lift was defined as % in the cluster versus in the cohort. Abbreviations: bpm, beats per minute; CAR-T, chimeric antigen receptor T-cell; CoNS, coagulase-negative Staphylococci; CRP, C-reactive protein; FN, febrile neutropenia; GNB, Gram-negative bacilli; MDR, multidrug-resistant; SBP, systolic blood pressure.

**Table 3:** Comparison of features included in the KAMILA model and bloodstream infection rates between development and validation cohorts.

	Development Cohort	Validation Cohort	*p*-Value
**N**	462	91	
**Haematological disease**, n (%)			
Lymphoma	131 (28.4)	26 (28.6)	1
Acute leukemia	218 (47.2)	40 (44.0)	0.65
**Clinical vital signs**, median [IQR]			
Temperature (°C)	38.3 [38.1–38.6]	38.5 [38.2–39.1]	<0.001
Heart rate (bpm)	103.0 [92.0–115.0]	107.0 [98.5–120.0]	0.02
SBP (mmHg)	99.0 [91.0–108.0]	98.0 [89.5–110.0]	0.65
**Laboratory findings**, median [IQR]			
Neutrophil count [×10^3^/µL]	0.1 [0–0.2]	0.1 [0–0.3]	0.59
CRP [mg/dL]	7.7 [3.7–18.8]	12.3 [6.3–20.7]	0.01
**Therapeutic interventions** ^a^, n (%)			
Cyclophosphamide	127 (27.5)	23 (25.3)	0.76
CAR-T	58 (12.6)	9 (9.9)	0.19
Blood transfusion	256 (55.4)	51 (56.0)	0.97
**Temporal hospital stays metrics, median [IQR]**			
Admission to onset of FN [days]	10.0 [3.0–18.8]	11.0 [4.0–20.5]	0.12
**Bloodstream infections**, n (%)			
Bloodstream infection	116 (25.1)	25 (27.5)	0.75
Bloodstream infection excluding CoNS	89 (19.3)	18 (19.8)	0.93
GNB related bloodstream infection	57 (12.3)	13 (14.2)	0.70
MDR-GNB related bloodstream infection	24 (5.2)	9 (9.9)	0.15

^a^ Administered from admission to episode start. Abbreviations: bpm, beats per minute; CAR-T, chimeric antigen receptor T-cell; CoNS, coagulase-negative Staphylococci; CRP, C-reactive protein; FN, febrile neutropenia; GNB, Gram-negative bacilli; MDR, multidrug-resistant; SBP, systolic blood pressure.

## Data Availability

The data that support the findings of this study are available from the corresponding author upon reasonable request.

## Supplementary Materials

The following supporting information can be downloaded at https://www.mdpi.com/article/10.3390/antibiotics14010013/s1, Table S1: Cluster-based distribution of pathogens in bloodstream infections in the development cohort; Table S2: Other bacterial isolates in non-blood cultures in the development cohort; Table S3: Comparative characteristics stratified by bloodstream infection status in the development cohort; Table S4: Cluster-based distribution of multidrug-resistant Gram-negative pathogens in bloodstream infections in the development cohort; Table S5: Cluster-based distribution of pathogens in bloodstream infections in the validation cohort; Table S6: Cluster-based distribution of multidrug-resistant Gram-negative pathogens in bloodstream infections in the validation cohort; Figure S1: Integrated density and boxplot representation of continuous features across clusters.

## References

[1] Tumbarello M., Spanu T., Caira M., Trecarichi E.M., Laurenti L., Montuori E., Fianchi L., Leone F., Fadda G., Cauda R. (2009). Factors Associated with Mortality in Bacteremic Patients with Hematologic Malignancies. Diagn. Microbiol. Infect. Dis..

[2] Gustinetti G., Mikulska M. (2016). Bloodstream Infections in Neutropenic Cancer Patients: A Practical Update. Virulence.

[3] El Omri H., Padmanabhan R., Taha R.Y., Kassem N., Elsabah H., Ellahie A.Y., Santimano A.J.J., Al-Maslamani M.A., Omrani A.S., Elomri A. (2024). Dissecting Bloodstream Infections in Febrile Neutropenic Patients with Hematological Malignancies, a Decade-Long Single Center Retrospective Observational Study (2009–2019). J. Infect. Public Health.

[4] Puerta-Alcalde P., Cardozo C., Marco F., Suárez-Lledó M., Moreno E., Morata L., Fernández-Avilés F., Gutiérrez-Garcia G., Chumbita M., Rosiñol L. (2020). Changing Epidemiology of Bloodstream Infection in a 25-Years Hematopoietic Stem Cell Transplant Program: Current Challenges and Pitfalls on Empiric Antibiotic Treatment Impacting Outcomes. Bone Marrow Transplant..

[5] Salas M.Q., Charry P., Puerta-Alcalde P., Martínez-Cibrian N., Solano M.T., Serrahima A., Nomdedeu M., Cid J., Lozano M., Chumbinta M. (2022). Bacterial Bloodstream Infections in Patients Undergoing Allogeneic Hematopoietic Cell Transplantation With Post-Transplantation Cyclophosphamide. Transplant. Cell. Ther..

[6] Ayaz C.M., Hazirolan G., Sancak B., Hascelik G., Akova M. (2022). Factors Associated with Gram-Negative Bacteremia and Mortality in Neutropenic Patients with Hematologic Malignancies in a High-Resistance Setting. Infect. Dis. Clin. Microbiol..

[7] Kern W.V., Roth J.A., Bertz H., Götting T., Dettenkofer M., Widmer A.F., Theilacker C., the Hospital Infection Surveillance System for Patients with Hematologic/Oncologic Malignancies Study Group (ONKO-KISS) (2019). Contribution of Specific Pathogens to Bloodstream Infection Mortality in Neutropenic Patients with Hematologic Malignancies: Results from a Multicentric Surveillance Cohort Study. Transpl. Infect. Dis..

[8] Guarana M., Nucci M., Nouér S.A. (2019). Shock and Early Death in Hematologic Patients with Febrile Neutropenia. Antimicrob. Agents Chemother..

[9] Averbuch D., Orasch C., Cordonnier C., Livermore D.M., Mikulska M., Viscoli C., Gyssens I.C., Kern W.V., Klyasova G., Marchetti O. (2013). European Guidelines for Empirical Antibacterial Therapy for Febrile Neutropenic Patients in the Era of Growing Resistance: Summary of the 2011 4th European Conference on Infections in Leukemia. Haematologica.

[10] Freifeld A.G., Bow E.J., Sepkowitz K.A., Boeckh M.J., Ito J.I., Mullen C.A., Raad I.I., Rolston K.V., Young J.-A.H., Wingard J.R. (2011). Clinical Practice Guideline for the Use of Antimicrobial Agents in Neutropenic Patients with Cancer: 2010 Update by the Infectious Diseases Society of America. Clin. Infect. Dis..

[11] Martinez-Nadal G., Puerta-Alcalde P., Gudiol C., Cardozo C., Albasanz-Puig A., Marco F., Laporte-Amargós J., Moreno-García E., Domingo-Doménech E., Chumbita M. (2020). Inappropriate Empirical Antibiotic Treatment in High-Risk Neutropenic Patients With Bacteremia in the Era of Multidrug Resistance. Clin. Infect. Dis..

[12] Chumbita M., Puerta-Alcalde P., Yáñez L., Cuesta M.A., Chinea A., Español Morales I., Fernández Abellán P., Gudiol C., Guerreiro M., González-Sierra P. (2022). Resistance to Empirical β-Lactams Recommended in Febrile Neutropenia Guidelines in Gram-Negative Bacilli Bloodstream Infections in Spain: A Multicentre Study. J. Antimicrob. Chemother..

[13] Chumbita M., Puerta-Alcalde P., Yáñez L., Angeles Cuesta M., Chinea A., Español-Morales I., Fernandez-Abellán P., Gudiol C., González-Sierra P., Rojas R. (2023). High Rate of Inappropriate Antibiotics in Patients with Hematologic Malignancies and Pseudomonas Aeruginosa Bacteremia Following International Guideline Recommendations. Microbiol. Spectr..

[14] Averbuch D., Tridello G., Hoek J., Mikulska M., Akan H., Yanez San Segundo L., Pabst T., Özçelik T., Klyasova G., Donnini I. (2017). Antimicrobial Resistance in Gram-Negative Rods Causing Bacteremia in Hematopoietic Stem Cell Transplant Recipients: Intercontinental Prospective Study of the Infectious Diseases Working Party of the European Bone Marrow Transplantation Group. Clin. Infect. Dis..

[15] Kara Ali R., Surme S., Balkan I.I., Salihoglu A., Sahin Ozdemir M., Ozdemir Y., Mete B., Can G., Ar M.C., Tabak F. (2020). An Eleven-Year Cohort of Bloodstream Infections in 552 Febrile Neutropenic Patients: Resistance Profiles of Gram-Negative Bacteria as a Predictor of Mortality. Ann. Hematol..

[16] Chumbita M., Puerta-Alcalde P., Gudiol C., Garcia-Pouton N., Laporte-Amargós J., Ladino A., Albasanz-Puig A., Helguera C., Bergas A., Grafia I. (2022). Impact of Empirical Antibiotic Regimens on Mortality in Neutropenic Patients with Bloodstream Infection Presenting with Septic Shock. Antimicrob. Agents Chemother..

[17] Paul M., Lador A., Grozinsky-Glasberg S., Leibovici L. (2014). Beta Lactam Antibiotic Monotherapy versus Beta Lactam-aminoglycoside Antibiotic Combination Therapy for Sepsis. Cochrane Database Syst. Rev..

[18] Mensa J., Barberán J., Soriano A., Llinares P., Marco F., Cantón R., Bou G., del Castillo J.G., Maseda E., Azanza J.R. (2018). Antibiotic Selection in the Treatment of Acute Invasive Infections by Pseudomonas Aeruginosa: Guidelines by the Spanish Society of Chemotherapy. Rev. Esp. Quim..

[19] Nouér S.A., Nucci M., Anaissie E. (2015). Tackling Antibiotic Resistance in Febrile Neutropenia: Current Challenges with and Recommendations for Managing Infections with Resistant Gram-Negative Organisms. Expert. Rev. Hematol..

[20] Pillinger K.E., Bouchard J., Withers S.T., Mediwala K., McGee E.U., Gibson G.M., Bland C.M., Bookstaver P.B. (2020). Inpatient Antibiotic Stewardship Interventions in the Adult Oncology and Hematopoietic Stem Cell Transplant Population: A Review of the Literature. Ann. Pharmacother..

[21] Gyssens I.C., Kern W.V., Livermore D.M. (2013). The Role of Antibiotic Stewardship in Limiting Antibacterial Resistance among Hematology Patients. Haematologica.

[22] Wolf J., Margolis E. (2020). Effect of Antimicrobial Stewardship on Outcomes in Patients With Cancer or Undergoing Hematopoietic Stem Cell Transplantation. Clin. Infect. Dis..

[23] Peiffer-Smadja N., Rawson T.M., Ahmad R., Buchard A., Georgiou P., Lescure F.-X., Birgand G., Holmes A.H. (2020). Machine Learning for Clinical Decision Support in Infectious Diseases: A Narrative Review of Current Applications. Clin. Microbiol. Infect..

[24] Lind M.L., Mooney S.J., Carone M., Althouse B.M., Liu C., Evans L.E., Patel K., Vo P.T., Pergam S.A., Phipps A.I. (2021). Development and Validation of a Machine Learning Model to Estimate Bacterial Sepsis Among Immunocompromised Recipients of Stem Cell Transplant. JAMA Netw. Open.

[25] Gallardo-Pizarro A., Peyrony O., Chumbita M., Monzo-Gallo P., Aiello T.F., Teijon-Lumbreras C., Gras E., Mensa J., Soriano A., Garcia-Vidal C. (2024). Improving Management of Febrile Neutropenia in Oncology Patients: The Role of Artificial Intelligence and Machine Learning. Expert. Rev. Anti-Infect. Ther..

[26] Foss A., Markatou M., Ray B., Heching A. (2016). A Semiparametric Method for Clustering Mixed Data. Mach. Learn..

[27] Peyrony O., Garcia-Pouton N., Chumbita M., Teijon-Lumbreras C., Aiello T.F., Monzó-Gallo P., Gallardo-Pizarro A., Ortiz-Maldonado V., Martinez-Cibrian N., Delgado J. (2024). Chimeric Antigen Receptor T-Cell Postinfusion Fever: Infection Profile, Clinical Parameters, and Biomarkers Trends to Assist Antibiotic Stewardship. Open Forum Infect. Dis..

[28] Mikulska M., Bartalucci C., Raiola A.M., Oltolini C. (2023). Does PTCY Increase Risk Infect?. Blood Rev..

[29] Garcia-Vidal C., Cardozo-Espinola C., Puerta-Alcalde P., Marco F., Tellez A., Agüero D., Romero-Santana F., Díaz-Beyá M., Giné E., Morata L. (2018). Risk Factors for Mortality in Patients with Acute Leukemia and Bloodstream Infections in the Era of Multiresistance. PLoS ONE.

[30] Garcia-Vidal C., Sanjuan G., Puerta-Alcalde P., Moreno-García E., Soriano A. (2019). Artificial Intelligence to Support Clinical Decision-Making Processes. EBioMedicine.

[31] Alali M., Mayampurath A., Dai Y., Bartlett A.H. (2022). A Prediction Model for Bacteremia and Transfer to Intensive Care in Pediatric and Adolescent Cancer Patients with Febrile Neutropenia. Sci. Rep..

[32] Sung L., Corbin C., Steinberg E., Vettese E., Campigotto A., Lecce L., Tomlinson G.A., Shah N. (2020). Development and Utility Assessment of a Machine Learning Bloodstream Infection Classifier in Pediatric Patients Receiving Cancer Treatments. BMC Cancer.

[33] Ahmad A., Khan S.S. (2019). Survey of State-of-the-Art Mixed Data Clustering Algorithms. IEEE Access.

[34] Albasanz-Puig A., Gudiol C., Puerta-Alcalde P., Ayaz C.M., Machado M., Herrera F., Martín-Dávila P., Laporte-Amargós J., Cardozo C., Akova M. (2021). Impact of the Inclusion of an Aminoglycoside to the Initial Empirical Antibiotic Therapy for Gram-Negative Bloodstream Infections in Hematological Neutropenic Patients: A Propensity-Matched Cohort Study (AMINOLACTAM Study). Antimicrob. Agents Chemother..

[35] Horan T.C., Andrus M., Dudeck M.A. (2008). CDC/NHSN Surveillance Definition of Health Care-Associated Infection and Criteria for Specific Types of Infections in the Acute Care Setting. Am. J. Infect. Control.

[36] Magiorakos A.-P., Srinivasan A., Carey R.B., Carmeli Y., Falagas M.E., Giske C.G., Harbarth S., Hindler J.F., Kahlmeter G., Olsson-Liljequist B. (2012). Multidrug-Resistant, Extensively Drug-Resistant and Pandrug-Resistant Bacteria: An International Expert Proposal for Interim Standard Definitions for Acquired Resistance. Clin. Microbiol. Infect..

[37] Miller J.M., Binnicker M.J., Campbell S., Carroll K.C., Chapin K.C., Gilligan P.H., Gonzalez M.D., Jerris R.C., Kehl S.C., Patel R. (2018). A Guide to Utilization of the Microbiology Laboratory for Diagnosis of Infectious Diseases: 2018 Update by the Infectious Diseases Society of America and the American Society for Microbiology. Clin. Infect. Dis..

[38] Drieux L., Brossier F., Sougakoff W., Jarlier V. (2008). Phenotypic Detection of Extended-Spectrum Beta-Lactamase Production in Enterobacteriaceae: Review and Bench Guide. Clin. Microbiol. Infect..

[39] Pierce V.M., Simner P.J., Lonsway D.R., Roe-Carpenter D.E., Johnson J.K., Brasso W.B., Bobenchik A.M., Lockett Z.C., Charnot-Katsikas A., Ferraro M.J. (2017). Modified Carbapenem Inactivation Method for Phenotypic Detection of Carbapenemase Production among Enterobacteriaceae. J. Clin. Microbiol..

[40] Giske C.G., Turnidge J., Cantón R., Kahlmeter G. (2022). On behalf of the EUCAST Steering Committee Update from the European Committee on Antimicrobial Susceptibility Testing (EUCAST). J. Clin. Microbiol..

[41] Buuren S., Groothuis-Oudshoorn C. (2011). MICE: Multivariate Imputation by Chained Equations in R. J. Stat. Softw..

[42] Foss A.H., Markatou M. (2018). Kamila: Clustering Mixed-Type Data in R and Hadoop. J. Stat. Soft..

[43] Kassambara A., Mundt F. (2020). Extract and Visualize the Results of Multivariate Data Analyses, R Package Factoextra Version 1.0.7. https://cran.r-project.org/web/packages/factoextra/index.html.

[44] Therneau T.M., Grambsch P.M. (2000). Modeling Survival Data: Extending the Cox Model.

[45] Ligges U., Maechler M. (2003). Scatterplot3d—An R Package for Visualizing Multivariate Data. J. Stat. Softw..

[46] Wickham H. (2016). Ggplot2.

[47] Sievert C. (2020). Interactive Web-Based Data Visualization with R, Plotly, and Shiny.

[48] Moons K.G.M., Altman D.G., Reitsma J.B., Ioannidis J.P.A., Macaskill P., Steyerberg E.W., Vickers A.J., Ransohoff D.F., Collins G.S. (2015). Transparent Reporting of a Multivariable Prediction Model for Individual Prognosis or Diagnosis (TRIPOD): Explanation and Elaboration. Ann. Intern. Med..

[49] Rahkonen M., Luttinen S., Koskela M., Hautala T. (2012). True Bacteremias Caused by Coagulase Negative Staphylococcus Are Difficult to Distinguish from Blood Culture Contaminants. Eur. J. Clin. Microbiol. Infect. Dis..

[50] Di Franco G. (2016). Multiple Correspondence Analysis: One Only or Several Techniques?. Qual. Quant..

[51] van Buuren S. (2018). Flexible Imputation of Missing Data.

[52] Preud’homme G., Duarte K., Dalleau K., Lacomblez C., Bresso E., Smaïl-Tabbone M., Couceiro M., Devignes M.-D., Kobayashi M., Huttin O. (2021). Head-to-Head Comparison of Clustering Methods for Heterogeneous Data: A Simulation-Driven Benchmark. Sci. Rep..

[53] Zhai Y., Bardel C., Vallée M., Iwaz J., Roy P. (2023). Performance Comparisons between Clustering Models for Reconstructing NGS Results from Technical Replicates. Front. Genet..

[54] Collins G.S., Dhiman P., Ma J., Schlussel M.M., Archer L., Calster B.V., Harrell F.E., Martin G.P., Moons K.G.M., van Smeden M. (2024). Evaluation of Clinical Prediction Models (Part 1): From Development to External Validation. BMJ.

[55] Altman D.G., Vergouwe Y., Royston P., Moons K.G.M. (2009). Prognosis and Prognostic Research: Validating a Prognostic Model. BMJ.

[56] Pagès J. (2014). Multiple Factor Analysis by Example Using R.

