# Supplementary material for: Development and Validation of a Machine Learning Model for the Prediction of Bloodstream Infections in Patients with Hematological Malignancies and Febrile Neutropenia
Source: Antibiotics (Basel). 2024 Dec 28;14(1):13. doi: 10.3390/antibiotics14010013 (PMC11760484; doi:10.3390/antibiotics14010013)
Supplement: Supplementary file 1 [file antibiotics-14-00013-s001.zip › antibiotics-3354953-supplementary.pdf]

SUPPLEMENTARY MATERIAL

Development and validation of a machine learning model for the prediction of bloodstream infections in patients with haematological malignancies and febrile neutropenia

Antibiotics

Antonio Gallardo-Pizarro, Christian Teijón-Lumbreras, Patricia Monzó-Gallo, Tommaso Francesco Aiello, Mariana Chumbita, Olivier Peyrony, Emmanuelle Gras, Cristina Pitart, Josep Mensa, Jordi Esteve, Alex Soriano, Carolina Garcia-Vidal

Corresponding Author: Dr. Carolina Garcia-Vidal. Department of Infectious Diseases, Hospital Clinic of Barcelona-IDIBAPS, Barcelona, Spain. C/ Villarroel 170, 08036 Barcelona, Spain. Tel: (+34) 93-227-5400 (ext. 2887). Email: cgarciav@clinic.cat

Supplementary Tables S1-6 p. 2-8

Supplementary Figure S1 p. 9

Supplementary Methods p. 10

TRIPOD Checklist: Prediction Model Development and Validation p. 11-12

SUPPLEMENARY TABLES

Table S1 Cluster-based distribution of pathogens in bloodstream infections in the development cohort

Cluster	Gram-positive BSIs		Gram-negative bacilli BSIs	
	**Pathogens**	**Number of Isolates**	**Pathogens**	**Number of Isolates**
**1**	CoNS	4		
	Rothia mucilaginosa	1		
**2**[*]	CoNS	8	*Escherichia coli*	8
	Enterococcus spp.	3	*Pseudomonas aeruginosa*	4
	Streptococus group	3	*Enterobacter* spp.	3
	Staphylococcus aureus	2	*Klebsiella pneumoniae*	2
	Brevibacterium spp.	1	*Citrobacter koseri*	1
	Corynebacterium spp.	1	*Morganella morganii*	1
			Proteus mirabilis	1
			Fusobacterium nucleatum	1
**3**	CoNS	26	*Escherichia coli*	16
	Enterococcus spp.	12	*Klebsiella pneumoniae*	12
	Streptococus group	10	*Pseudomonas aeruginosa*	7
	Gemella haemolysans	2	*Pseudomonas* spp.	2
	Brevibacterium spp.	1	*Stenotrophomonas maltophilia*	2
	Rothia mucilaginosa	1	*Capnocytophaga sputigena*	2
	Micrococcus spp.	1	*Fusobacterium nucleatum*	2
	Kocuria rhizophila	1	*Sphingomonas* spp.	1
	Clostridium ramosum	1	*Enterobacter* spp.	1
			Kingella kingae	1
			Leptotrichia trevisanii	1

[*]Note: In addition to the bacterial pathogens listed, this cluster also includes two cases of fungemia caused by *Candida* spp.
Abbreviations: **BSI**, bloodstream infection; **CoNS**, coagulase-negative Staphylococci

Table S2 Other bacterial isolates in non-blood cultures in the development cohort

	Cluster 1		Cluster 2		Cluster 3	
**Respiratory tract**	Mycobacterium tuberculosis complex	1	*Pseudomonas aeruginosa*	3		
			Staphylococcus aureus	1		
**Urine**	*Enterococcus* spp.	2	*Enterococcus* spp.	4	*Enterococcus* spp.	5
	Enterobacter spp.	1	*Escherichia coli***	3	*Escherichia coli***	1
			Streptococcus pneumoniae	2	*Klebsiella pneumoniae***	1
			*Klebsiella pneumoniae***	1	*Lactobacillus* spp.	1
			Lactobacillus spp.	1		
			Legionella pneumophila	1		
**Stools**	*Campylobacter* spp.	2	*Clostridium difficile*	6	*Clostridium difficile*	2
	Shigella spp/ EIEC	1	*Campylobacter* spp.	3		
			Salmonella spp.	2		
			Shigella spp/ EIEC	1		
**Others***	*Mycobacterium tuberculosis* complex	1	*Pseudomonas aeruginosa*	3	*Pseudomonas aeruginosa*	1
			Staphylococcus aureus	1	*Enterococcus* spp.	1
			Escherichia coli	1		
			CoNS	1		

*The 'Others' category includes a variety of specimens, such as blood, cerebrospinal fluid, skin or tissue biopsies, among others
**Each bacterium includes one case of ESBL
Abbreviation: **CoNS**, coagulase-negative Staphylococci; **EIEC**, Enteroinvasive *Escherichia coli*; **ESBL**, extended-spectrum beta-lactamase

Table S3 Comparative characteristics stratified by bloodstream infection status in the development cohort

Variable	Bloodstream Infection*				*p-value*	Missing data
	Yes		No			
**N**	116		346			
Female, n (%)	41	(35.2)	141	(40.8)	0.36	0
Age (years), median [IQR]	55.5	[45-68]	59	[44-68]	0.78	0
**Co-morbidities, n (%)**						
Diabetes mellitus	9	(7.8)	40	(11.6)	0.32	0
Chronic lung disease	22	(19)	41	(11.8)	0.08	0
Chronic heart disease	26	(22.4)	91	(26.3)	0.48	0
Chronic kidney disease	7	(6)	19	(5.5)	0.83	0
Chronic liver disease	10	(8.6)	21	(6.1)	0.46	0
Solid neoplasm	25	(22.4)	59	(17.1)	0.25	0
HIV	2	(1.7)	2	(1.0)	0.57	0
**Previous admission, n (%)**						
Within 1 month	26	(22.4)	89	(25.7)	0.56	0
Within 3 months	40	(34.5)	134	(38.7)	0.48	0
**Previous ICU admission, n (%)**						
Within 1 month	3	(2.5)	14	(4)	0.66	0
Within 3 months	9	(7.8)	29	(8.4)	0.99	0
Previous insolation of MDR, n (%)	13	(11.2)	42	(12.1)	0.92	0
**Temporal hospital stays metrics, median [IQR]**						
Admission to onset of FN (days)	26	[13-5-31]	2	[1-4.8]	<0.001	0
**Hematologic disease, n (%)**						
Lymphoma	19	(16.4)	112	(32.4)	0.001	0
Acute leukemia	71	(61.2)	147	(42.5)	<0.001	0
Multiple Myeloma	10	(8.6)	38	(11.0)	0.59	0
Myelodysplastic syndrome	18	(15.5)	30	(8.7)	0.06	0
**Vital signs at FN onset, median [IQR]**						
Temperature (°C)	38.3	[38.1-38.7]	38.3	[38.1-38.6]	0.07	0
Heart rate (bpm)	103	[90-120]	102	[93-114]	0.45	0
SBP (mmHg)	97	[85.5-117]	100	[92-109]	0.04	5
DBP (mmHg	57	[51-63.8]	59	[52-65]	0.17	5
Respiratory rate (breaths per minute)	18	[18-20]	18	[18-20]	0.85	67
Oxygen saturation (%)	96	[94.2-97]	96	[94-97]	0.59	0
**Laboratory Parameters, median (IQRs)**						
Neutrophil count [cells/mm^3]	0	[0-0.1]	0.1	[0-0.3]	<0.001	0
Glucose [mg/dL]	109	[97.8-124.3]	103	[92-118]	0.06	38

Lactate [mmol/L]	16.6	[9.9-26.2]	14.2	[11.4-22.6]	0.93	420
LDH [U/L]	163.5	[120.5-222]	193	[141.8-303.2]	0.01	146
CRP [mg/dL]	8.7	[4.4-17.8]	7.4	[3.4-13.9]	0.14	112
Creatinine [mg/dL]	0.7	[0.5-0.9]	0.7	[0.6-0.9]	0.28	36
ALT [U/L]	24.5	[15.8-52]	23	[14-46]	0.55	116
ALP [U/L]	17	[12.8-25.3]	19	[14-31]	0.07	115
Albumin [g/L]	35.5	[32.8-37.3]	35	[33-38]	0.96	303
**Hematopoietic stem cell transplant, n (%)**						
Post-admission	53	(45.7)	142	(41)[a]	0.44	0
**Chemotherapeutic exposure, n (%)[**]**						
Cyclophosphamide[a]	47	(40.5)	80	(30.1)	<0.001	0
PTCY-based prophylaxis for GVHD	24	(45.2)[b]	40	(28.2)[b]	0.02	0
**CAR-T therapy, n (%)**						
Post-admission	2	(1.7)	56	(16.2)	<0.001	0
**Corticosteroid therapy, n (%)**						
Within 1 month	16	(13.8)	48	(13.9)	0.98	0
Within 3 months	28	(24.8)	79	(22.8)	0.87	0
**Antibiotic exposure, n (%)**						
Prior antibiotic therapy (within 1 month)	25	(21.6)	79	(22.8)	0.88	0
**Corticosteroid therapy, n (%)**						
Within 1 month	16	(13.8)	48	(13.9)	0.98	0
Within 3 months	28	(24.8)	79	(22.8)	0.87	0
**Blood product administration, n (%)**						
Admission-to-episode blood transfusion	76	(65.5)	180	(52.0)	0.01	0

[*]Two cases of fungemia caused by *Candida glabrata* and *Candida albicans* were included in the analysis

[**]54 different chemotherapy treatments and multiple treatment regimens have been studied. Those with a frequency below 10% were excluded; for improved interpretation, they were not included in this table

[a]Administered from admission to episode start

[b]Percentages were calculated within the total number of patients treated with cyclophosphamide. *Abbreviations*: **CAR-T**, chimeric antigen receptor T-cell; **CRP**, C-reactive protein; **SBP**, systolic blood pressure; **PTCY**, post-transplant cyclophosphamide; **HIV**, human immunodeficiency virus; **ICU**, intensive care unit; **MDR**, multidrug-resistant; **DBP**, diastolic blood pressure; **LDH**, lactate dehydrogenase; **ALP**, alkaline phosphatase; **IQR**, interquartile range; **ALT**, alanine aminotransferase; **GVHD**, graft-versus-host disease

Table S4 Cluster-based distribution of multidrug-resistant Gram-negative pathogens in bloodstream infections in the development cohort

**Cluster***	**Resistance Type**	**Pathogens**	**Number of Isolates**
**2**	Extended-Spectrum Beta-Lactamase	*Escherichia coli*	2
		Enterobacter spp.	1
	Carbapenemase	*Morganella morganii*	1
	No Fermenting Gram-Negative Bacilli	*Pseudomonas aeruginosa*	2
**3**	Extended-Spectrum Beta-Lactamase	*Escherichia coli*	5
		Klebsiella pneumoniae	4
	ESBL-Carbapenemase	*Klebsiella pneumoniae*	4
		Escherichia coli	1
		Enterobacter spp.	1
	No Fermenting Gram-Negative Bacilli	*Pseudomonas aeruginosa*	2
		Stenotrophomonas maltophilia	2

*Note: Cluster 1 is not represented in the table as there were no isolates of these pathogens in this cluster

Table S5 Cluster-based distribution of pathogens in bloodstream infections in the validation cohort

Cluster	Gram-positive BSIs		Gram-negative bacilli BSIs	
	**Pathogens**	**Number of Isolates**	**Pathogens**	**Number of Isolates**
1	*Enterococcus* spp.	1		
2*	CoNS	3	*Escherichia coli*	2
	Enterococcus spp.	1	*Pseudomonas aeruginosa*	1
3	CoNS	3	*Escherichia coli*	4
	Streptococcus group	2	*Stenotrophomonas maltophilia*	3
	Staphylococcus aureus	1	*Pseudomonas aeruginosa*	1
	Enterococcus spp.	1	*Serratia marcescens*	1
			Enterobacter spp.	1
			Bacteroides thetaiotaomicron	1

*Note: In addition to the bacterial pathogens listed, this cluster also includes one case of fungemia caused by *Candida tropicalis*
Abbreviations: **BSI**, bloodstream infection; **CoNS**, coagulase-negative Staphylococci

Table S6 Cluster-based distribution of multidrug-resistant Gram-negative pathogens in bloodstream infections in the validation cohort

**Cluster***	**Resistance Type**	**Pathogens**	**Number of Isolates**
**2**	Extended-Spectrum Beta-Lactamase	*Escherichia coli*	1
	No Fermenting Gram-Negative Bacilli	*Pseudomonas aeruginosa*	1
**3**	Extended-Spectrum Beta-Lactamase	*Escherichia coli*	3
	No Fermenting Gram-Negative Bacilli	*Stenotrophomonas maltophilia*	3
		Pseudomonas aeruginosa	1

*Note: Cluster 1 is not represented in the table as there were no isolates of these pathogens in this cluster

SUPPLEMENARY FIGURE

Figure S1 Integrated density and boxplot representation of continuous features across clusters

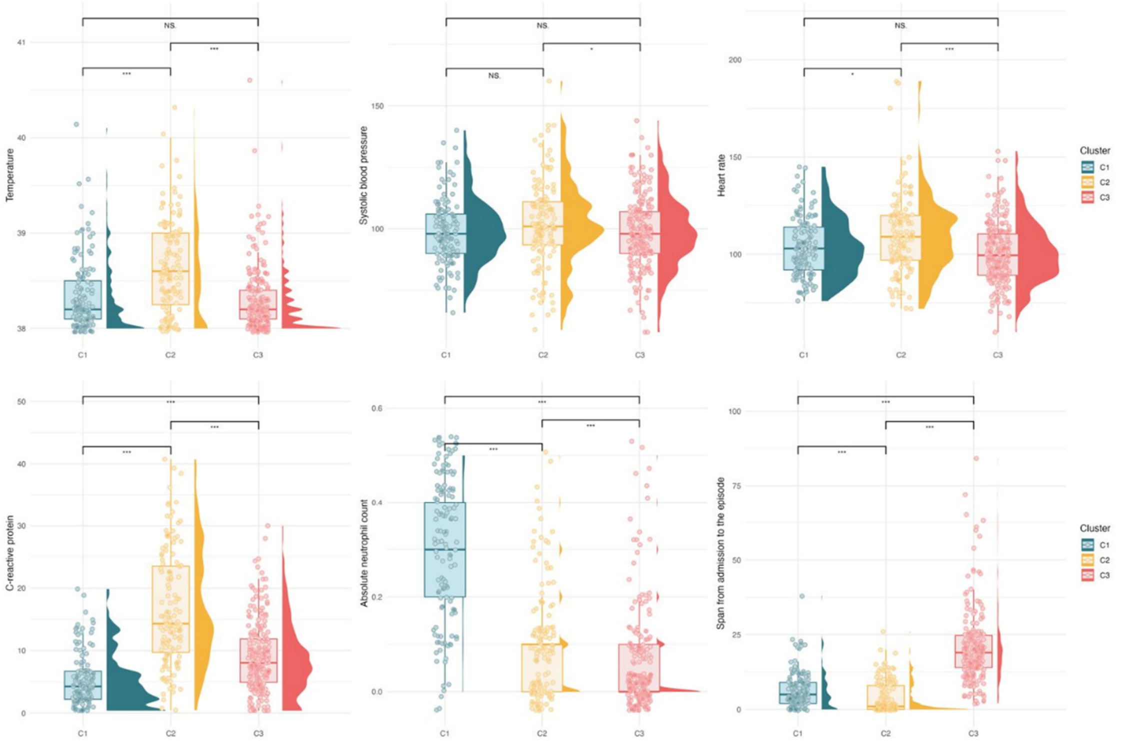

The X-axis represents the number of clusters, while the Y-axis denotes the values of a specific continuous feature. Post-hoc paired comparisons were conducted using Dunn's test with Bonferroni correction. Legend: NS = Not Significant, * = $p < 0.05$, *** = $p < 0.001$

SUPPLEMENTARY METHODS

Cluster Analysis

- The number of clusters was supported using the prediction strength criterion.
- The KAMILA algorithm was evaluated with three, four, and five clusters.
- Based on prediction strength, the three-cluster model (0.539) outperformed the four- and five-cluster solutions (0.404 and 0.296, respectively).

This approach ensured an optimal balance between model fit and complexity, favoring the three-cluster solution.

TRIPOD Checklist: Prediction Model Development and Validation.

Section/Topic	Item*		Checklist Item	Page
Title and abstract				
Title	1	D;V	Identify the study as developing and/or validating a multivariable prediction model, the target population, and the outcome to be predicted.	1
Abstract	2	D;V	Provide a summary of objectives, study design, setting, participants, sample size, predictors, outcome, statistical analysis, results, and conclusions.	1
Introduction				
Background and objectives	3a	D;V	Explain the medical context (including whether diagnostic or prognostic) and rationale for developing or validating the multivariable prediction model, including references to existing models.	1-2
	3b	D;V	Specify the objectives, including whether the study describes the development or validation of the model or both.	2
Methods				
Source of data	4a	D;V	Describe the study design or source of data (e.g., randomized trial, cohort, or registry data), separately for the development and validation data sets, if applicable.	8
	4b	D;V	Specify the key study dates, including start of accrual; end of accrual; and, if applicable, end of follow-up.	8
Participants	5a	D;V	Specify key elements of the study setting (e.g., primary care, secondary care, general population) including number and location of centres.	8
	5b	D;V	Describe eligibility criteria for participants.	8
	5c	D;V	Give details of treatments received, if relevant.	ST3
Outcome	6a	D;V	Clearly define the outcome that is predicted by the prediction model, including how and when assessed.	9-10
	6b	D;V	Report any actions to blind assessment of the outcome to be predicted.	9-10
Predictors	7a	D;V	Clearly define all predictors used in developing or validating the multivariable prediction model, including how and when they were measured.	9-10
	7b	D;V	Report any actions to blind assessment of predictors for the outcome and other predictors.	9-10

Sample size	8	D;V	Explain how the study size was arrived at.	8
Missing data	9	D;V	Describe how missing data were handled (e.g., complete-case analysis, single imputation, multiple imputation) with details of any imputation method.	9
Statistical analysis methods	10a	D	Describe how predictors were handled in the analyses.	9-10
	10b	D	Specify type of model, all model-building procedures (including any predictor selection), and method for internal validation.	9-10
	10c	V	For validation, describe how the predictions were calculated.	9-10
	10d	D;V	Specify all measures used to assess model performance and, if relevant, to compare multiple models.	10
	10e	V	Describe any model updating (e.g., recalibration) arising from the validation, if done.	10
Risk groups	11	D;V	Provide details on how risk groups were created, if done.	10
Development vs. validation	12	V	For validation, identify any differences from the development data in setting, eligibility criteria, outcome, and predictors.	6, 9-10
Results				
Participants	13a	D;V	Describe the flow of participants through the study, including the number of participants with and without the outcome and, if applicable, a summary of the follow-up time. A diagram may be helpful.	2-3
	13b	D;V	Describe the characteristics of the participants (basic demographics, clinical features, available predictors), including the number of participants with missing data for predictors and outcome.	ST3
	13c	V	For validation, show a comparison with the development data of the distribution of important variables (demographics, predictors and outcome).	6
Model development	14a	D	Specify the number of participants and outcome events in each analysis.	3-5
	14b	D	If done, report the unadjusted association between each candidate predictor and outcome.	3-5
Model specification	15a	D	Present the full prediction model to allow predictions for individuals (i.e., all regression coefficients, and model intercept or baseline survival at a given time point).	3-4
	15b	D	Explain how to the use the prediction model.	4
Model performance	16	D;V	Report performance measures (with CIs) for the prediction model.	3-4
Model-updating	17	V	If done, report the results from any model updating (i.e., model specification, model performance).	N/A
Discussion				
Limitations	18	D;V	Discuss any limitations of the study (such as nonrepresentative sample, few events per predictor, missing data).	7-8
Interpretation	19a	V	For validation, discuss the results with reference to performance in the development data, and any other validation data.	7
	19b	D;V	Give an overall interpretation of the results, considering objectives, limitations, results from similar studies, and other relevant evidence.	7-8
Implications	20	D;V	Discuss the potential clinical use of the model and implications for future research.	8
Other information				
Supplementary information	21	D;V	Provide information about the availability of supplementary resources, such as study protocol, Web calculator, and data sets.	11
Funding	22	D;V	Give the source of funding and the role of the funders for the present study.	11

* 'D' denotes items relevant only to the development of a prediction model, while 'V' indicates items pertaining solely to validation. Items applicable to both are marked as 'D;V'.
Abbreviations: **ST2**, supplementary table S2; **ST3**, supplementary table S3.
